# Anthropometrical Features of Para-Footballers According to Their Cerebral Palsy Profiles and Compared to Controls

**DOI:** 10.3390/ijerph17239071

**Published:** 2020-12-04

**Authors:** José M. Sarabia, Carmen Doménech, Enrique Roche, Néstor Vicente-Salar, Raul Reina

**Affiliations:** 1 Sports Research Centre, Department of Sport Sciences, Miguel Hernández University, 03202 Elche, Spain; jsarabia@umh.es (J.M.S.); carmendomenechribes@live.com.mx (C.D.); 2 Alicante Institute for Health and Biomedical Research (ISABIAL Foundation), 03010 Alicante, Spain; eroche@umh.es (E.R.); nvicente@umh.es (N.V.-S.); 3 Department of Applied Biology-Nutrition, Institute of Bioengineering, Miguel Hernandez University, 03202 Elche, Spain; 4 CIBER Fisiopatología de la Obesidad y Nutrición (CIBEROBN), Instituto de Salud Carlos III (ISCIII), 28029 Madrid, Spain

**Keywords:** body composition, paralympics, para-sport, brain impairment, soccer, football

## Abstract

Cerebral palsy (CP) football is a team para-sport practiced by para-athletes with eligible impairments of hypertonia, athetosis, and ataxia. This study aimed: (1) to describe the anthropometrical and body composition profiles of international CP para-footballers with different CP profiles (i.e., spastic diplegia, athetosis/ataxia, spastic hemiplegia, and minimum impairment); (2) to analyze the differences between both affected/nondominant and nonaffected/dominant sides; and (3) to compare the sample of international-level CP para-footballers (*n* = 141) with a sample of highly trained able-bodied footballers (*n* = 39). Anthropometric measures included four breadths, nine girths, and six skinfolds, while body composition was measured through fat mass (including Carter’s, Faulkner’s, and Withers’ equations), muscle mass (Lee’s equation), and bone mass (Rocha’s and Martin’s equations). This study found differences between the able-bodied footballers and the following impairment profiles: spastic diplegia (skinfolds); ataxia/athetosis (corrected calf of the nondominant side, and calf skinfolds for both sides); and spastic hemiplegia (all measurements excepting femur breadth, and thigh and ankle girths). No differences were found between para-athletes with minimum impairment and the able-bodied footballers. This study demonstrates that football players with or without physical impairments of hypertonia athetosis or ataxia may be considered homogeneous in shape when dominant size is compared. Besides, the study provides reference scores on anthropometric measures and body composition of international-level CP para-footballers that can help sports coaches and physical trainers to monitor physical fitness of their para-athletes.

## 1. Introduction

In the field of sports, the assessment of body composition is an essential factor because it has been related to performance and even to success in a specific sport, in combination with other factors such as technical, tactical, physical, and psychological skills [1,2]. In the case of football, body composition is among the key fitness elements to football players’ performance not only in adults but also in young football players [3]. While fat-free mass has been strongly correlated to strength and power performance [4], body fat might increase the injury risk [5] and negatively influence players’ performance [6]. Hence, for football staff and researchers, a better understanding of the determinants of success—such as the specific anthropometric characteristics of the players—may be crucial for training and talent identification. Previous studies on able-bodied football players have disclosed significant differences in anthropometric and fitness measures between playing levels [7], across playing positions, and between age categories [8].

The body composition and anthropometrical characteristics of the para-athletes have also been previously described and in some cases related to performance in swimming [9,10,11], blind sports [11,12], track and field [11,13,14], wheelchair sports [15,16,17], or rowing [18]. However, most of these previous studies included a mixed pool of para-athletes with different types of impairment and from different para-sports. In addition, to the best of the authors’ knowledge, the previous studies with cerebral palsy (CP) athletes only described the basic anthropometry measures (i.e., height, weight, and body mass index) [19,20] or somatotype [21]. Only the study by Yanci et al. [22] compared anthropometry measures with the physical performance (i.e., jump capacity), but body composition and anthropometrical characteristics of CP football players have not been previously described and compared to able-bodied football players.

CP football is a seven-a-side modality of football, played by ambulant athletes with CP or acquired brain injury. Para-footballers are classified into sport classes giving a special relevance to their CP profile and impairment severity. Over the last decades, CP football has used a functional classification system for their para-athletes developed by the Cerebral Palsy International Sports and Recreation Association (CPISRA) [23]. Specifically, those with moderate spastic diplegia, moderate athetoid or ataxic profile, and moderate spastic hemiplegia are grouped in FT5, FT6, or FT7, respectively. In addition, the mild forms of these impairments—also called “minimum impairment criteria” to be eligible for competing in this team para-sport—are classified together in the FT8 sport class [24].

The literature shows that both children and adults with CP tend to have below-average weight, linear growth, muscle mass, and fat mass compared with their peers [25,26,27]. Indeed, people with hemiplegia might show an increased bone loss and muscle atrophy, especially on the hemiplegic side [28] and more common in the upper body [29]. It has also been suggested that exercise may modify or reverse skeletal muscle abnormalities [30].

Knowing the anthropometric attributes of highly trained CP football players in relation to impairment/sport classes would provide the basis upon which practitioners could provide individualized practice, in an attempt to evaluate and develop the specific attributes and optimize players’ performance. Thus, the aims of this study were (1) to describe the anthropometrical and body composition profiles of international CP football players for each CP profile; (2) to analyze the differences between both affected/nondominant and nonaffected/dominant sides; and (3) to compare them with a sample of highly trained able-bodied football players.

## 2. Materials and Methods

### 2.1. Participants

One hundred and forty-one footballers (age = 24.8 ± 6.3 years) with more than seven years of experience in football participated in the study (Table 1). Of those, 102 were international para-footballers from different countries who participated at the 2013 CPISRA Intercontinental Cup (Barcelona, Spain), a qualifying tournament for the 2015 CP-Football World Championships. These players were classified as spastic diplegia (*n* = 8), athetosis/ataxia (*n* = 14), spastic hemiplegia (*n* = 64), or minimum impairment (*n* = 16). The rest of the players were the control group (CG), that is, a group of 39 able-bodied footballers who were playing in the third Spanish football division. Prior to involvement in the investigation, all participants gave written informed consent after a detailed written and oral explanation of the potential risks and benefits resulting from participation in this study, as outlined in the Declaration of Helsinki (2013). Approval by the institutional review board (Office for Projects Evaluation, OEP) was obtained before the study began (Ref. DPS.RRV0.01.14).

### 2.2. Anthropometric Determinations

All variables were measured by a Level 2 anthropometrist certified by the International Society for the Advancement of Kinanthropometry (ISAK) with an individual technical error of measurement (TEM) of 0.76–0.39% for skinfolds and 0.12% for the remaining parameters. The errors were considered acceptable for ISAK standards (<7.5% for skinfolds and <1.5% for the remaining measurements). All measurements were made following the guidelines stated by ISAK [31] except for chest skinfold, which was according to Heyward and Stollarczyk [32]. The limb measurements were obtained for both body sides (except for neck girth and abdominal skinfold) in all the participants and taken in duplicate. An average of the two measurements was recorded.

The total body mass of each participant was measured in kilograms using a Tanita digital scale (model BC-601), breadths with a Holtain bicondylar caliper (Holtain, Crosswell, UK), girths with a metallic nonextensible tape (Lufkin, Sparks, NV, USA), and skinfolds with a Holtain Tanner/Whitehouse skinfold caliper (Holtain, Crosswell, UK). The following four breadths were measured: humerus, wrist, femur, and ankle. Regarding girths, six were measured: relaxed arm; flexed and tensed arm; neck; thigh; medial calf; and ankle. Finally, seven skinfolds were also measured: triceps (Tr); chest (Ch); subscapular (Sb); supraspinale (Sp); abdominal (A); thigh (Th); and medial calf (Ca). In addition, the corrected arm, thigh, and calf were calculated using the formula:

Corrected Girth = Girth − (π Skinfold)(1)

### 2.3. Body Composition

Three-component models of body composition were used, dividing fat-free mass into lean tissue mass and bone mineral content. Due to specific equations to calculate the different body mass types not having been developed for para-athletes with CP or other related neurological conditions, the equations recommended for athletes to calculate the components of body mass have been used [33]. Percentage of body fat mass was calculated using three different methods: Yuhasz’s equation modified by Faulkner [34], Carter’s equation [35], and calculating the body density with Withers’ equations [36] and converting to body fat percentage using Siri’s equation [37]. Percentage of bone mass was calculated according to two different equations: Rocha’s [31] and Martin’s [38] equations. Percentage of muscle mass was calculated from Lee’s equation [39]. In addition, the following sums were considered for fat content calculations [40]:

- Three skinfolds
  ∑3Sk = Ch + A + Th(2)
- Six skinfolds
  ∑6Sk = Tr + Sb + Sp + A + Th + Ca(3)
- Upper body skinfolds
  ∑UpSk = Tr + Sb(4)
- Lower body skinfolds
  ∑LowSk = Th + Ca(5)

### 2.4. Body Proportionality

Body proportionality analyses were conducted using the Phantom stratagem proposed by Ross and Wilson [41], which has been previously applied in other sports with able-bodied athletes [42,43]. The Phantom is a unisex, bilaterally symmetrical conceptual model that was derived from reference data of men and women [44]. The Phantom-Z scores (Z-Scores) for each anthropometric variable were used to demonstrate the number and direction of standard deviations that each of the groups varied against the Phantom. Each variable was transformed in a Z-Score adjusting it to the Phantom size using the following equation:

Z-Score = (1/*s*) *v* [(170.18/*h*)*^d^ − P*] (6)

where *v* is the size of any variable, 170.18 is the Phantom height constant, *h* is the subject’s height, *d* is a dimensional exponent, *P* is the Phantom value for variable *v*, and *s* is the Phantom standard deviation value. These Z-Scores have a 0 mean, so a Z-Score higher than 0 means that the subject is proportionally greater than the Phantom, and Z-score lower than 0 means the opposite. This allows data standardization, providing a reference profile for each type of impairment and allowing future comparisons of individual scores with the results of this study.

### 2.5. Statistical Analyses

Statistical analysis was performed using the Statistical Package for Social Sciences (SPSS Inc., version 240.0 for Windows, Chicago, IL, USA). Statistical significance was set at the α-level of 0.05 for two-tailed tests. Distribution of the data was tested by using the Kolmogorov–Smirnov and the Shapiro–Wilk tests, and the Q–Q plot.

The results indicated that data were not normally distributed for the whole group or for the CP profiles (i.e., sport classes). For this reason, the median as central tendency measure and the interquartile range (25th and 75th percentiles) as a measure of the spread of the data have been considered in this study. Therefore, the nonparametric Wilcoxon test was used to detect significant differences between dominant and nondominant sides.

In addition, differences among the CP profiles were identified using the Kruskal–Wallis test and the Bonferroni correction in case of significant findings between groups. Finally, Friedman’s comparisons were used to compare body fat mass equations and body bone mass equation between them inside each CP profile. When we found differences in Friedman’s comparisons, we performed the Wilcoxon matched-pairs test for multiple analysis with Bonferroni’s correction. Practical significance was assessed by calculating effect size (*d*) [45], according to the values suggested by Cohen [46]: above 0.8, between 0.8 and 0.50, between 0.50 and 0.2, and lower than 0.2 were considered as large, moderate, small, and trivial, respectively.

## 3. Results

### 3.1. Dominant vs. Nondominant Sides

Comparisons between the dominant and nondominant sides are shown in Table 2 and Table 3. While spastic diplegia, minimum impairment, and control groups showed trivial differences in some anthropometrical data, the athetosis/ataxia group showed differences in girths, and the spastic hemiplegia group showed marked differences in all measures except for the trunk skinfolds.

### 3.2. Comparison with the Control Group

**Figure 1:**
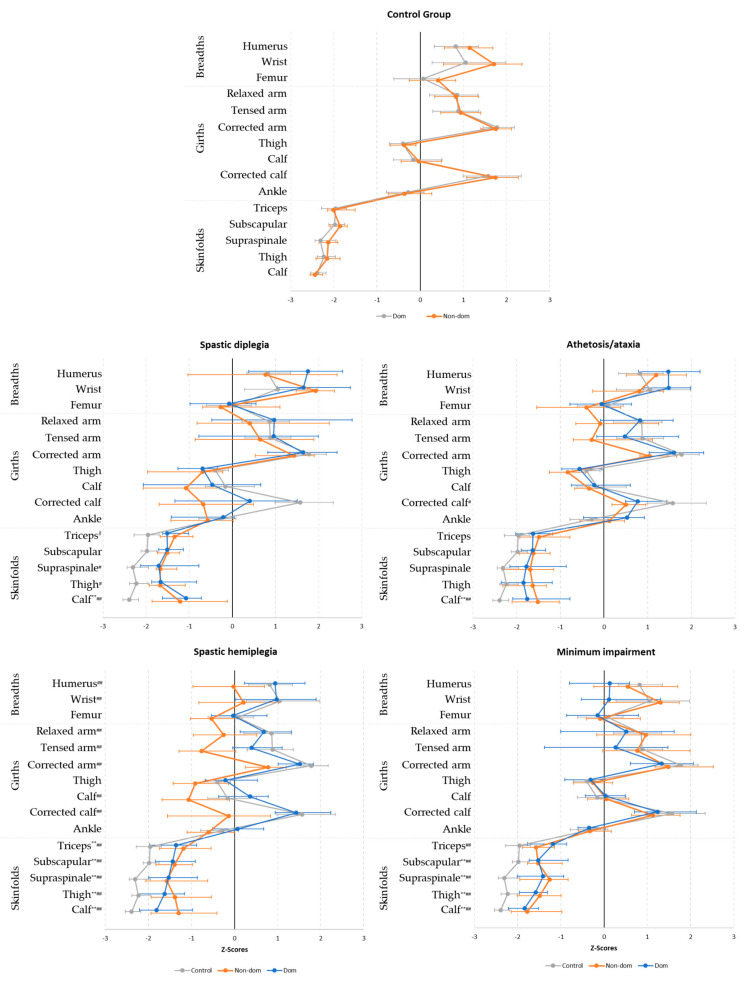
Z-Scores for dominant (dom) and nondominant (non-dom) sides of the body for each group and their comparison from the control group. ** significant difference between the control group and the dominant side *p* < 0.01, ^#^ significant difference between the control group and the nondominant side *p* < 0.05, ^##^ significant difference between the control group and the nondominant side *p* < 0.01.

The comparisons between each CP group and the control group were made comparing Z-Scores. The Z-Scores are shown in Figure 1 and fully detailed in Table S1. All CP groups show differences in skinfolds of both sides of the body with the CG. In addition, the spastic hemiplegia group showed differences with the CG on many of the breadths and girths for the nondominant side.

### 3.3. Equation Comparison

Regarding body fat mass equations, Faulkner’s equation was significantly different from Withers’ and Carter’s equations for spastic diplegia (*Z* = 2.52, *p* = 0.012, *d* = 0.89, for both comparisons), minimum impairment (*Z* = 2.95 and 3.52, *p* < 0.001, *d* = 0.74 and 0.88, respectively) and control group (*Z* = 5.43 and 5.44, *p* < 0.001, *d* = 0.87), while in the athetosis/ataxia group, it was significantly different only from Carter (*Z* = 3.11, *p* = 0.002, *d* = 0.83), and in the spastic hemiplegia group, all equations were significantly different from the others (*Z* = 5.74–6.94, *p* < 0.0001, *d* = 0.72–0.87).

Regarding body bone mass equations, both Rocha’s and Martin’s equations were significantly different for each group (spastic diplegia, *Z* = 2.52, *p* = 0.012, *d* = 0.89; athetosis/ataxia, *Z* = 3.30, *p* = 0.001, *d* = 0.88; spastic hemiplegia, *Z* = 6.96, *p* < 0.001, *d* = 0.87; minimum impairment, *Z* = 3.52, *p <* 0.001, *d* = 0.88; CG, *Z* = 5.44, *p* < 0.001, *d *= 0.87).

## 4. Discussion

Anthropometric and body composition parameters are of top importance in determining energy requirements [47] and are related with performance in sports [4]. However, there are still few studies describing body composition characteristics of athletes with a varied impairment and from different para-sports. The difficulty in collecting data is due to the different classes and degrees of impairment, which makes it challenging to establish a representative sample. Therefore, the main contributions of the current study are the descriptive anthropometrical data and proportionality characteristics for each impairment profile for elite CP footballers.

Previous studies have reported a broad range of fatness and leanness in adults with CP [26,48]. However, these studies have used a small and heterogeneous (i.e., different motor impairments and ambulation status) sample of sedentary people with CP, and in most cases with children. Only a few studies have studied the body composition of athletes with CP, but in most cases they mixed athletes with other different eligible impairments (e.g., vision impairment, limb deficiency), and they did not compare the results of para-athletes with an able-bodied group [10,11,14]. To the best of the authors’ knowledge, only two previous studies have included only elite athletes with CP [13,21] but only Runciman [13] compared the six para-athletes included with a control group. Like this study, our results suggest that the anthropometrical measures and body composition of players with CP were closely matched to the able-bodied players in anthropometrical measures and body composition estimations. Although some differences in the affected/nondominant side appeared, these results are close to the average population (i.e., Z-Scores between −2 and 2).

In contrast with the fatness found in previous studies [26,48], all CP subgroups in the current study showed higher skinfolds than the CG. The authors hypothesize that this could be due to different nutritional habits and grades of training between groups [49]. While all players in the CG group were professional or semi-professional football players and included a considerable amount of off-field training (e.g., strength and aerobic training at the gym) and in some cases a nutritional controlled planning, the CP footballers were from different countries and, in some of them, this para-sport is not yet professionalized. In addition, the amount of the off-field training was in some cases reduced, mainly due to the impairment. Overall, able-bodied players were 6 kg heavier and 9 cm taller on average than the CP footballers, but with similar BMI for all groups (from 21.9 to 23.4 kg·m^2^) and in the ranges showed by other studies [21]. In addition, the profiles for both types of footballers were similar in appearance when the dominant sizes were matched proportionally (Figure 1). This indicates that, whether through training or self-selection, specific proportionality characteristics dominate the preferred morphology for elite football players. The main difference (i.e., the body fat mass measure trough skinfolds) could be justified by the professionalism in physical preparation as shown by Ackland et al. [50] in canoe and kayak paddlers.

Moving to comparison for dominant and nondominant sides, any asymmetry was found in able-bodied footballers as supporting previous studies [51]. In addition, only one anthropometric measure was different between sides for the spastic diplegia subgroup (i.e., humerus breadth). This reveals the homogeneity of the impairment for both sides in these para-athletes. Even though this group showed a similar profile in proportionality to the CG, showing differences only in skinfolds, it is possible to see a high dispersion of calf data on both sides. While the median (25th and 75th percentiles) of the CG for the corrected calf was 1.74 (10.08, 2.27), dominant and nondominant sides of the spastic diplegia group were 0.4 (−1.34, 1.51) and −0.68 (−1.7, 0.49), respectively. This reflects the atrophy of the calf muscles [52]. However, the proportionality profile of girths in the lower limbs were like the CG despite the impairment, showing a similar effect of training in lower body. Still, the low number of players in this group and the high heterogeneity of the impairment prevent these data from showing a clear difference with the able-bodied players. However, the rest of the measures showed a very similar profile to the CG.

The athetosis/ataxia group showed higher anthropometric measures in the upper body (i.e., wrist breadth and relaxed, tensed, and corrected arm girth) and lower body (i.e., thigh, calf, and ankle girths) for the dominant side than for the nondominant side. Consequently, body muscle and bone mass percentages were higher when they were calculated with the dominant-side measures. However, no differences between sides were found for skinfolds and body fat mass percentage. The difficulty of the players with athetosis or ataxia to control their muscle tone and to reduce involuntary muscle activity [53] could be a consequence of this difference. Thus, we can see that the differences between tensed and relaxed arm for the dominant and nondominant sides were 1.3 cm and 1.8 cm, respectively, while it was higher and more homogeneous for both sides in the other groups. These increased girths result in a higher percentage of muscle mass when it is calculated for the dominant side of the body. When this group is compared with the CG, only corrected calf (for nondominant side) and calf skinfolds for both sides showed significant differences, suggesting that they may be due to the different training regimen between groups.

The spastic hemiplegia group showed the highest differences between sides of all groups. As expected, all anthropometrical measures were different between dominant and nondominant sides except the trunk skinfolds (i.e., subscapular, chest, and supraspinale skinfolds). Hence, estimations of body fat, muscle, and bone percentages were higher for the dominant side than for the nondominant side. By contrast, Runciman et al. [13] found symmetry in fat and bone mass in elite paralympic track sprinters with hemiplegia. However, they included only five athletes with hemiplegia and the tendency raises the suspicion of a trend to be different when the data are normalized by age (e.g., femoral neck Z-score was 0.40 ± 0.63 and 00.05 ± 0.91 for nonaffected and affected, respectively) [13]. In addition, the literature had shown a lower total bone and fat-free mass and a similar fat mass—measured with dual-energy X-ray absorptiometry—in people with CP compared with controls [54], reducing these differences when athletes with hemiplegia were compared with a control group [13]. In our results, the nondominant side of those with unilateral spasticity showed smaller breadths, girths, and muscle mass, and higher skinfolds and fat mass than the CG, but the dominant side was higher only in fat mass (i.e., skinfolds). Like previous studies [13], even though some measures were different from the CG, the Z-Scores of the current study were near to 0, showing a similar profile of average population. It was proposed that the differences reported in these individuals with CP were the result of low volumes of ambulation, and subsequent lower bone and muscle loading [55]. Hence, participation in sport activity could reduce these differences with able-bodied people.

Only one anthropometric measure was different between sides for the minimum impairment group (i.e., femur breadth). Even though this group was composed of players with different CP profiles, they showed no differences with the CG either for the dominant or the nondominant side. This reflects the low impact of impairment on these players, showing similar anthropometrical profiles to those of able-bodied football players. These results are in line with previous studies which compared the performance of this group of players with minimum impairment with a control group, showing similar sports performance [56], but also when comparing para-footballers with the different CP profiles included in this study [21].

The combination of these findings has an important implication for the necessity for people with CP to perform exercise [55]. It seems that elite athletes with CP who have undertaken physical exercise over many years may achieve similar adaptations to able-bodied athletes from the same sport. However, the finding that the anthropometrical measures were lower on the affected areas in these groups of athletes indicate that there may be an upper limit for the adaptations that occur and, as the differences showed by the minimum impairment and the other groups, this limit seems dependent on the grade of impairment.

Some limitations should be mentioned. Although the number of players with cerebral palsy included in the study is a good representation of elite CP footballers even for each impairment, the sample size of some groups is too small to achieve high statistical power. In addition, the equations used to estimate the body composition have not been previously validated in people with CP. For those reasons, results should be interpreted with caution.

## 5. Conclusions

This study was conducted with para-athletes from 12 different national teams that took part in a world-level competition. It has been demonstrated that there are no major differences between a group of able-bodied football players and the moderately impaired CP football sport profiles when the dominant size is measured and the data are relativized to the player’s height beyond the fat mass. No differences were found between those para-athletes belonging to the sport class categorized as minimum (i.e., mild) impairment and the able-bodied football players. When comparing body sides, the most common profile in this para-sport (i.e., spastic hemiplegia) [57] was the group with more significant differences between dominant (i.e., nonaffected) and nondominant (i.e., affected) body sides, that is, all the variables measured excepting trunk skinfolds (i.e., subscapular, chest, and supraspinale skinfolds). Besides, this study demonstrates that football players with or without a physical impairment (i.e., hypertonia, athetosis, or ataxia) may be considered homogeneous in shape when dominant size is compared. This reflects the importance of measuring both or dominant side in people with CP and not only the right side as the recommendations for general populations say [31]. In addition, we provide reference scores of anthropometric measures and body composition scores of international-level CP footballers that can help sports coaches and physical trainers to monitor the physical fitness of their para-athletes. This study provides a proportionality profile to compare para-athletes’ anthropometry which could help, for example, in the selection process—choosing players with the best match with the elite profile—or monitoring the evolution of players’ body composition. Coaches would also monitor and guide their athletes’ training to achieve the reference values of elite para-footballers.

## Figures and Tables

**Table 1:** Characteristics of the sample and general body measurements.

Variable	Total Sample	Spastic Diplegia	Athetosis/Ataxia	Spastic Hemiplegia	Minimum Impairment	Control Group
*n*	141	8	14	64	16	39
Laterality (R/L)	77/64	3/5	8/6	24/40	9/7	33/6
Ethnicity (CA/AF)	135/6	8/0	13/1	62/2	16/0	36/3
Training experience (year)	11 (6, 16)	12 (5, 15)	10 (10, 12)	10 (5, 14)	17 (5, 22)	15 (15, 16)
Age (year)	23 (20, 29)	23.5 (18, 33.3)	24.5 (19.8, 34)	23 (21, 30)	26.5 (21.3, 38.3)	22 (20, 23)
Weight (kg)	70.9 (64, 76.7)	63.4 (60.1, 73.3) ^†^	68.1 (62.2, 73)	67.3 (62.4, 75) ^††^	73.6 (69.4, 78.7) ^†^	74.5 (69.3, 80)
Height (cm)	176 (172, 181)	171.5 (168.3, 178.8)	173 (170.8, 179.3) ^†^	174 (172, 178.9) ^††^	179 (170.4, 183.8)	181 (176, 184)
BMI (kg·m^−2^)	22.6 (21.5, 23.9)	21.9 (19.7, 24.4)	22.5 (20.4, 23.9)	22.1 (21, 23.7)	23.4 (21.6, 25.6)	23.2 (22.1, 24)

R = the right leg is dominant; L = the left leg is dominant; CA = Caucasian; AF = Afro-American. Data are delivered as median (25th and 75th percentiles). ^†^ significant difference with the control group *p* < 0.05; ^††^ significant difference with the control group *p* < 0.01.

**Table 2:** Anthropometric measures and body composition of the four CP profiles and the able-bodied football players (control group).

	Spastic Diplegia (*n* = 8)		Athetosis/Ataxia (*n* = 14)		Spastic Hemiplegia (*n* = 64)		Minimum Impairment (*n* = 16)		Control Group (*n* = 39)	
	Dom	Non-Dom	Dom	Non-Dom	Dom	Non-Dom	Dom	Non-Dom	Dom	Non-Dom
Breadths (cm)										
Humerus	7.2 (6.8, 7.4) *	7 (6.4, 7.3)	7 (6.8, 7.5)	7.1 (6.8, 7.3)	7 (6.6, 7.3) **	6.6 (6.4, 6.9)	7 (6.6, 7.5)	6.9 (6.4, 7.2)	7.2 (7, 7.5) *	7.2 (6.9, 7.4)
Wrist	5.9 (5.6, 6.1)	5.9 (5.7, 6)	5.7 (5.5, 6.1) **	5.5 (5.2, 5.7)	5.6 (5.4, 5.9) **	5.4 (5.1, 5.6)	5.9 (5.5, 6)	5.6 (5.4, 5.8)	6 (5.8, 6.2)	5.9 (5.5, 6.1)
Femur	9.5 (9.3, 10)	9.7 (9.6, 10)	9.7 (9.4, 10)	9.7 (9.2, 9.8)	9.8 (9.5, 10.1) **	9.5 (9.3, 9.8)	10 (9.7, 10.3) **	9.9 (9.5, 10.1)	10.2 (10, 10.5) **	10.1 (9.7, 10.4)
Ankle	7.5 (7.3, 7.8)	7.4 (7.1, 7.5)	7.5 (7.3, 7.7)	7.5 (7.2, 7.8)	7.4 (7.2, 7.8) **	7.2 (7, 7.5)	7.4 (7.2, 7.8)	7.4 (7.1, 7.7)	7.6 (7.2, 8)	7.6 (7.2, 8)
Girths (cm)										
Relaxed arm	29.2 (26.3, 33.3)	27.8 (26.1, 32)	29.3 (27.6, 30.9) **	27.7 (26.3, 29.1)	29.6 (27.3, 30.6) **	26.9 (25.3, 28.6)	30 (28.1, 32.6)	29 (25.7, 32.9)	30.2 (29.3, 31.7)	30.2 (28.9, 32.1)
Flexed arm	32.4 (28.7, 33.8)	30.7 (28.7, 33.8)	30.6 (30.3, 33.7) *	29.5 (28.8, 31.7)	31.2 (29.8, 32.6) **	28.7 (27.4, 30.1)	33.3 (30.7, 34.7)	31.8 (27.8, 35)	33.3 (32.3, 34.8)	33.2 (31.3, 34.6)
Neck	370.0 (360.0, 37.9)		36.8 (35.4, 37.8)		36.5 (35.2, 37.7)		36.4 (35.9, 38.4)		37.2 (35.6, 38.3)	
Thigh	49.9 (49.6, 53.9)	50.2 (45.4, 52.6)	51.7 (49.8, 54.1) *	50.7 (48.3, 53.5)	53.7 (51.3, 56.3) **	50.6 (47.9, 53.7)	54.9 (52.7, 55.8)	54.2 (52.7, 55.2)	54.4 (53.3, 55.7)	54.2 (51.6, 56.2)
Calf	34.5 (31.5, 37.3)	32.5 (31.3, 35.7)	35.6 (35, 36.9) *	35.1 (34.2, 36)	36.8 (35, 38.2) **	34.2 (32.1, 35.7)	37.1 (36.1, 37.9)	37 (35.7, 38.6)	37.3 (35.9, 38.7)	37.3 (35.7, 38.8)
Ankle	21.4 (20.3, 22.7)	21 (20.6, 22.4)	22.7 (21.8, 23.2) *	22.2 (21.4, 22.6)	22.3 (21.4, 23.4) **	21.5 (20.7, 22.3)	22.3 (21.6, 23.2)	22.6 (21.3, 23)	22.7 (21.9, 23.1)	22.5 (21.9, 23.4)
Corrected arm	26.1 (23.5, 29.4)	25.1 (22.6, 27.2)	26.3 (24.9, 28) *	24.5 (23.9, 25.5)	26 (24.8, 27.7) **	23.5 (21.9, 24.9)	26.7 (25.5, 29.9)	26.2 (22.6, 29.8)	28 (26.5, 29.1)	28.2 (26.5, 29.9)
Corrected thigh	45.9 (42.6, 48.7)	44.3 (41, 47.3)	48 (46.4, 50)	47.2 (44.5, 48.9)	49.1 (47.1, 51.4) **	44.8 (43.1, 47.7)	50.2 (47.2, 51.1)	49.7 (47.6, 52.1)	51.5 (49.3, 52.4)	50.8 (49, 52.9)
Corrected calf	31.1 (28.2, 34.2)	28.7 (27.2, 31.9)	32.7 (31.7, 33.9)	31.9 (31.5, 33.4)	34 (32.6, 35.5) **	30.6 (28.1, 33.2)	34.8 (32.3, 35.4)	34.2 (33.1, 36.7)	35.8 (34.4, 36.9) *	35.3 (33.7, 37.1)
Skinfolds (mm)										
Triceps	8.8 (8.3, 10.8)	9.4 (8.1, 11.9)	8.5 (6.5, 10.1)	9.4 (7.1, 12.1)	9.3 (6.7, 11.9) **	10.4 (7.6, 13.6)	8.9 (7.2, 11.1)	9.9 (8.1, 12)	6.9 (6.3, 8.7)	7 (5.3, 8.4)
Subscapular	9.6 (8.9, 11.5)	9.9 (8.3, 11.1)	8.9 (7.8, 10.6)	9.2 (7.6, 11.3)	10 (8.2, 12.8)	10.2 (8.2, 12.5)	9.8 (8.7, 12.5)	10.2 (8.9, 13.3)	8.5 (7.1, 9.2)	7.6 (6.9, 8.6)
Chest	6.5 (5.6, 9.2)	7 (6.5, 7.5)	6.7 (5.8, 11.2)	7.2 (4.5, 10.9)	7.9 (5.7, 11.7)	8.1 (5.1, 11.8)	8.3 (6, 12)	8.4 (5.6, 11.3)	4.2 (2.9, 4.9)	4.1 (3.1, 4.6)
Supraspinale	7.7 (6.1, 120.0)	8.1 (7.6, 9.8)	7.7 (6.1, 11.3)	7.9 (5, 10.4)	8.6 (6.6, 11.7)	8.6 (6.7, 13.2)	9.9 (7.2, 12.4)	9.7 (6.5, 11.8)	6.3 (5.1, 7.4) *	5.6 (4.8, 7.1)
Abdominal	17.8 (11.5, 20.8)		15.4 (9, 25.2)		18 (10.2, 26.2)		19.3 (12.6, 29.7)		9.2 (7.4, 10.3)	
Thigh	13.4 (11.4, 200.0)	13.1 (11, 18.9)	11.9 (7.6, 17.3)	13.9 (8.1, 16.1)	13.7 (8.5, 17.7) **	15.8 (11.1, 22.4)	15 (10.5, 19.8)	14.7 (11.1, 16.6)	9.3 (7.6, 12)	9 (7.6, 11.1)
Calf	11.2 (8.5, 12.6)	10.5 (7.5, 15.5)	7.9 (6.6, 12.6)	9.2 (6.2, 11.4)	7.7 (5.9, 12) **	10.1 (7.1, 14.2)	7.7 (6.1, 12)	7.7 (6, 9.5)	4.9 (4.2, 5.9)	5.3 (4.3, 6.4)

Data are delivered as median (25th and 75th percentiles); Dom = dominant side of the body; Non-Dom = nondominant side of the body. * significant difference with the nondominant side *p* < 0.05, ** significant difference with the nondominant side *p* < 0.01.

**Table 3:** Body composition of the four CP profiles and the able-bodied football players (control group).

	Spastic Diplegia (*n* = 8)		Athetosis/Ataxia (*n* = 14)		Spastic Hemiplegia (*n* = 64)		Minimum Impairment (*n* = 16)		Control Group (*n* = 39)	
	Dom	Non-Dom	Dom	Non-Dom	Dom	Non-Dom	Dom	Non-Dom	Dom	Non-Dom
Fat mass (%)										
Carter’s equation	9.6 (9, 11.6)	9.6 (9.3, 10.1)	9 (7.7, 11.3)	9.2 (7.6, 12.5)	9.7 (7.7, 120.0) **	10.5 (8.2, 13.1)	10.8 (8.9, 12.2)	10.4 (8.6, 11.9)	7.5 (6.6, 8.2)	7.1 (6.6, 8.1)
Faulkner’s equation	12.7 (11.1, 13.8)	12.4 (11.7, 13.2)	12.1 (10.7, 14.5)	12.5 (10.2, 14.7)	13.1 (10.9, 15.2) **	13.4 (10.9, 15.5)	13.6 (11.7, 15.2)	13.3 (11.8, 15.8)	10.6 (9.9, 11.2) *	10.3 (9.6, 11.2)
Withers’ equation	10 (9, 14)	10.2 (9.6, 12.2)	10 (8.1, 12.5)	9.8 (7.9, 13.8)	10.8 (8.3, 140.0) **	11.7 (8.7, 15.1)	11.9 (9.2, 16.1)	10.7 (9.2, 14.6)	6.7 (6.1, 7.6)	6.7 (6.1, 7.5)
∑3 Sk	36.1 (32.1, 51.2)	37.6 (34.6, 38.5)	37.4 (27.4, 50.2)	36.4 (23.1, 52.2)	38.9 (27.5, 54.4) **	41.1 (28.2, 57.9)	43.7 (33.8, 61.3)	41.1 (32.7, 56.5)	23 (18.9, 26.7)	22 (18.7, 26.1)
∑6 Sk	67.1 (60.7, 85.6)	66.4 (63.9, 71.4)	61.2 (49.1, 82.6)	63.3 (47.6, 94)	67.7 (48.8, 89.3) **	75.3 (53.6, 100.2)	78.4 (60.3, 91.6)	74.2 (57.6, 88.3)	46.5 (38.2, 53.1)	42.8 (38.5, 52.9)
∑Upper body Sk	18.7 (16.9, 20.9)	19 (18.2, 19.8)	17.4 (14, 19.9)	18.8 (14.8, 22.6)	19.6 (15.3, 25.7) **	21 (17.1, 25.8)	18.7 (17.6, 22.6)	21 (18.3, 24.1)	15.2 (13.9, 17.5) *	14.9 (12.6, 16.6)
∑Lower body Sk	24.1 (19.6, 33.8)	24.7 (19.9, 30)	19.8 (14.8, 32.7)	23.7 (15.7, 28.2)	22.1 (14.5, 31.2) **	25.7 (18.2, 36.3)	25.5 (16.5, 32)	23 (16.8, 25.1)	14.2 (12.2, 18.4)	14 (12.4, 16.2)
Muscle mass (%)										
Lee’s equation	43.7 (42.2, 45.4)	41.3 (39.5, 43.6)	43.8 (41.9, 46.8) *	41.5 (38.9, 45.8)	44.8 (42.8, 47.9) **	39 (36, 42.3)	42.9 (41, 43.9)	41.8 (40.1, 43.3)	45.8 (44.4, 47.2)	45.5 (44.1, 47.2)
Bone mass (%)										
Rocha’s equation	17.3 (16.7, 19.8)	17.6 (16.5, 19.7)	17.6 (16, 18.2) *	16.5 (15.3, 17.8)	17.5 (16, 18.6) **	16.3 (15.4, 17.4)	17.1 (16.1, 18.1) *	16.5 (15.2, 17.4)	17.6 (16.9, 18.4) *	17.1 (16.3, 17.9)
Martin’s equation	14.4 (13.6, 15.4)	13.7 (12.8, 14.7)	13.6 (13.3, 14.6) *	13.5 (13.1, 14.1)	13.9 (12.6, 15) **	12.7 (12, 13.5)	13.7 (12.4, 14.3) **	12.3 (11.8, 13.5)	13.8 (13.3, 14.4) *	13.6 (12.8, 14)

Data are delivered as median (25th and 75th percentiles); Dom = dominant side of the body; Non-Dom = nondominant side of the body; ∑3 Sk = the sum of chest, abdominal, and thigh skinfolds; ∑6 Sk = the sum of triceps, subscapular, supraspinale, abdominal, thigh, and calf skinfolds; ∑Upper body Sk = the sum of triceps and subscapular skinfolds; ∑Lower body Sk = the sum of thigh and calf skinfolds. * significant difference with the nondominant side *p* < 0.05, ** significant difference with the nondominant side *p* < 0.01.

## Supplementary Materials

The following are available online at https://www.mdpi.com/1660-4601/17/23/9071/s1, Table S1: Z-Scores for dominant (dom) and nondominant (non-dom) sides of the body for each group and their comparison from the control group.
