# Supplementary material for: Anthropometrical Features of Para-Footballers According to Their Cerebral Palsy Profiles and Compared to Controls
Source: Int J Environ Res Public Health. 2020 Dec 4;17(23):9071. doi: 10.3390/ijerph17239071 (PMC7730112; doi:10.3390/ijerph17239071)
Supplement: Supplementary file 1 [file ijerph-17-09071-s001.zip › Supplemental Table 1.docx]

|  | **Spastic diplegia (n = 8)** | | **Athetosis/ataxia (n = 14)** | | **Spastic hemiplegia (n = 64)** | | **Minimum impairment (n = 16)** | | **Control Group (n = 39)** | |
| --- | --- | --- | --- | --- | --- | --- | --- | --- | --- | --- |
|  | **Dom** | **Non-dom** | **Dom** | **Non-dom** | **Dom** | **Non-dom** | **Dom** | **Non-dom** | **Dom** | **Non-dom** |
| Weight | -0,23 (-1,15, 0,63) | | -0,11 (-0,76, 0,48) | | -0,25 (-0,64, 0,54) | | -0,16 (-0,52, 0,71) | | -0,15 (-0,45, 0,19) | |
| Breadths |  |  |  |  |  |  |  |  |  |  |
| Humerus | 1,75 (0,38, 2,56) | 0,77 (-1,03, 2,43) | 1,48 (0,79, 2,2) | 1,19 (0,52, 1,89)^##^ | 0,94 (0,24, 1,64) | -0,03 (-0,97, 0,7)^††^ | 0,55 (-0,24, 1,71) | 0,14 (-0,8, 0,6) | 1,15 (0,56, 1,68) | 0,82 (0,33, 1,35) |
| Wrist | 1,65 (1,04, 2,74) | 1,94 (1,48, 2,37)^##^ | 1,48 (0,9, 1,99) | 0,81 (-0,25, 1,37) | 0,98 (0,03, 1,9) | 0,21 (-0,83, 1,03)^††^ | 1,31 (-0,02, 1,74) | 0,12 (-0,53, 1,31) | 1,71 (0,54, 2,36) | 1,05 (0,28, 1,98) |
| Femur | -0,08 (-0,98, 0,55) | -0,27 (-0,69, 1,1) | -0,06 (-0,78, 0,63) | -0,4 (-1,54, 0,38) | -0,04 (-0,54, 0,76) | -0,54 (-1,03, 0,17) | -0,09 (-0,41, 0,84) | -0,15 (-0,87, 0,8) | 0,41 (-0,25, 0,82) | 0,07 (-0,61, 0,44) |
| Girths |  |  |  |  |  |  |  |  |  |  |
| Relaxed arm | 0,97 (-0,48, 2,78) | 0,4 (-0,82, 2,25) | 0,82 (-0,08, 1,58) | -0,09 (-0,64, 1,26) | 0,68 (0,13, 1,32) | -0,26 (-0,96, 0,51)^††^ | 0,97 (-0,17, 2,01) | 0,52 (-1,01, 1,62) | 0,82 (0,34, 1,35) | 0,86 (0,22, 1,33) |
| Flexed arm | 0,96 (-0,78, 2) | 0,65 (-0,86, 1,89) | 0,48 (-0,17, 1,71) | -0,29 (-0,7, 1,11) | 0,4 (-0,04, 1,1) | -0,77 (-1,29, 0,03)^††^ | 0,77 (-0,03, 1,99) | 0,27 (-1,38, 1,48) | 0,94 (0,47, 1,4) | 0,88 (0,29, 1,36) |
| Neck | 0,82 (0,01, 1,57) | | 0,52 (-0,25, 1,84) | | 0,4 (-0,23, 1,19) | | 0,06 (-0,6, 1,23) | | 0,02 (-0,67, 0,73) | |
| Thigh | -0,69 (-1,26, -0,34) | -0,7 (-1,97, -0,22) | -0,56 (-0,96, -0,04) | -0,83 (-1,26, -0,42) | -0,22 (-0,69, 0,53) | -0,92 (-1,43, -0,14) | -0,25 (-0,71, 0,2) | -0,32 (-0,91, 0,03) | -0,37 (-0,7, -0,1) | -0,4 (-0,71, -0,09) |
| Calf | -0,47 (-2,07, 0,66) | -1,08 (-2,07, -0,29) | -0,23 (-0,74, 0,6) | -0,34 (-0,67, -0,02) | 0,36 (-0,36, 0,78) | -1,07 (-1,68, -0,12)^††^ | 0,06 (-0,38, 0,57) | 0,03 (-0,43, 0,49)^#^ | -0,03 (-0,44, 0,49) | -0,16 (-0,62, 0,51) |
| Ankle | -0,21 (-1,42, 0,05) | -0,58 (-1,43, 0,02) | 0,53 (-0,47, 0,93) | 0,12 (-0,55, 0,47) | 0,08 (-0,5, 0,68) | -0,61 (-1,1, 0) | -0,33 (-0,6, 0,16) | -0,35 (-0,6, 0,03) | -0,36 (-0,74, 0,27) | -0,28 (-0,78, 0,09) |
| Corrected arm | 1,64 (0,83, 2,43) | 1,43 (0,53, 1,9) | 1,6 (1,04, 2,28) | 1,04 (0,83, 1,67) | 1,52 (1,01, 1,83) | 0,79 (0,26, 1,2)^††^ | 1,49 (1,18, 2,52) | 1,33 (0,61, 2,07) | 1,75 (1,4, 2,12) | 1,78 (1,45, 2,18) |
| Corrected calf | 0,4 (-1,34, 1,51) | -0,68 (-1,7, 0,49)^††^ | 0,77 (0,49, 1,44) | 0,51 (0,18, 0,96)^†^ | 1,44 (0,94, 2,24) | -0,13 (-1,55, 0,83)^††^ | 1,13 (0,64, 1,76) | 1,24 (0,71, 2,13)^##^ | 1,74 (1,08, 2,27) | 1,57 (0,99, 2,34) |
| Skinfolds |  |  |  |  |  |  |  |  |  |  |
| Triceps | -1,52 (-1,62, -1,01) | -1,35 (-1,66, -0,91)^†^ | -1,63 (-2,02, -1,18) | -1,49 (-1,93, -0,78) | -1,36 (-1,98, -0,88)^††^ | -1,19 (-1,75, -0,54)^††^ | -1,57 (-1,89, -1,15) | -1,18 (-1,77, -0,87)^††^ | -2,01 (-2,15, -1,51) | -1,96 (-2,28, -1,7) |
| Subscapular | -1,51 (-1,71, -1,14) | -1,51 (-1,74, -1,22) | -1,64 (-1,89, -1,34) | -1,61 (-1,93, -1,24) | -1,44 (-1,84, -0,91)^††^ | -1,39 (-1,83, -0,97)^††^ | -1,53 (-1,77, -0,97)^†^ | -1,53 (-1,73, -0,83)^††^ | -1,86 (-2,09, -1,69) | -1,98 (-2,12, -1,75) |
| Supraspinale | -1,71 (-2,13, -0,78) | -1,68 (-1,77, -1,29)^†^ | -1,78 (-2,15, -0,87) | -1,69 (-2,33, -1,15) | -1,53 (-1,99, -0,86)^††^ | -1,57 (-2,06, -0,62)^††^ | -1,26 (-1,95, -0,83)^††^ | -1,41 (-2,01, -0,93)^††^ | -2,13 (-2,34, -1,91) | -2,31 (-2,44, -1,95) |
| Abdominal | -0,99 (-1,86, -0,58)^††^ | | -1,3 (-2,12, -0,11)^††^ | | -1,03 (-1,96, -0,14)^††^ | | -0,96 (-1,83, 0,58)^††^ | | -2,14 (-2,38, -1,99) | |
| Thigh | -1,67 (-1,88, -0,83) | -1,68 (-1,93, -1,1)^†^ | -1,85 (-2,35, -1,19) | -1,63 (-2,32, -1,32) | -1,63 (-2,25, -1,16)^††^ | -1,39 (-1,93, -0,54)^††^ | -1,49 (-2,01, -1)^††^ | -1,59 (-1,96, -1,31)^†^ | -2,16 (-2,41, -1,86) | -2,23 (-2,38, -1,96) |
| Calf | -1,08 (-1,62, -0,72)^††^ | -1,21 (-1,87, -0,12)^††^ | -1,76 (-2,09, -0,78)^††^ | -1,52 (-2,1, -1,03)^††^ | -1,81 (-2,21, -0,97)^††^ | -1,3 (-1,94, -0,41)^††^ | -1,78 (-2,14, -0,98)^††^ | -1,84 (-2,22, -1,51)^†^ | -2,44 (-2,55, -2,26) | -2,39 (-2,54, -2,18) |

**Table S1.** Z-Scores for dominant (dom) and non-dominant (non-dom) sides of the body for each group and their comparison from the control group

Data are delivered as median (25th and 75th percentiles); Dom = Dominant side of the body; Non-dom = Non-dominant side of the body; ** significant difference between the control group and the dominant side *p* < .01, # significant difference between the control group and the non-dominant side *p* < .05, ## significant difference between the control group and the non-dominant side *p* < .01.
